# MR Image Analytics to Characterize the Upper Airway Structure in Obese Children with Obstructive Sleep Apnea Syndrome

**DOI:** 10.1371/journal.pone.0159327

**Published:** 2016-08-03

**Authors:** Yubing Tong, Jayaram K. Udupa, Sanghun Sin, Zhengbing Liu, E. Paul Wileyto, Drew A. Torigian, Raanan Arens

**Affiliations:** 1 Medical Image Processing Group, Department of Radiology, University of Pennsylvania, Philadelphia, Pennsylvania, United States of America; 2 Division of Respiratory and Sleep Medicine, Children’s Hospital at Montefiore, Bronx, New York, United States of America; 3 Department of Biostatistics and Epidemiology, University of Pennsylvania, Philadelphia, Pennsylvania, United States of America; Technion—Israel Institute of Technology, ISRAEL

## Abstract

**Purpose:**

Quantitative image analysis in previous research in obstructive sleep apnea syndrome (OSAS) has focused on the upper airway or several objects in its immediate vicinity and measures of object size. In this paper, we take a more general approach of considering all major objects in the upper airway region and measures pertaining to their individual morphological properties, their tissue characteristics revealed by image intensities, and the 3D architecture of the object assembly. We propose a novel methodology to select a small set of salient features from this large collection of measures and demonstrate the ability of these features to discriminate with very high prediction accuracy between obese OSAS and obese non-OSAS groups.

**Materials and Methods:**

Thirty children were involved in this study with 15 in the obese OSAS group with an apnea-hypopnea index (AHI) = 14.4 ± 10.7) and 15 in the obese non-OSAS group with an AHI = 1.0 ± 1.0 (p<0.001). Subjects were between 8–17 years and underwent T1- and T2-weighted magnetic resonance imaging (MRI) of the upper airway during wakefulness. Fourteen objects in the vicinity of the upper airways were segmented in these images and a total of 159 measurements were derived from each subject image which included object size, surface area, volume, sphericity, standardized T2-weighted image intensity value, and inter-object distances. A small set of discriminating features was identified from this set in several steps. First, a subset of measures that have a low level of correlation among the measures was determined. A heat map visualization technique that allows grouping of parameters based on correlations among them was used for this purpose. Then, through T-tests, another subset of measures which are capable of separating the two groups was identified. The intersection of these subsets yielded the final feature set. The accuracy of these features to perform classification of unseen images into the two patient groups was tested by using logistic regression and multi-fold cross validation.

**Results:**

A set of 16 features identified with low inter-feature correlation (< 0.36) yielded a high classification accuracy of 96% with sensitivity and specificity of 97.8% and 94.4%, respectively. In addition to the previously observed increase in linear size, surface area, and volume of adenoid, tonsils, and fat pad in OSAS, the following new markers have been found. Standardized T2-weighted image intensities differed between the two groups for the entire neck body region, pharynx, and nasopharynx, possibly indicating changes in object tissue characteristics. Fat pad and oropharynx become less round or more complex in shape in OSAS. Fat pad and tongue move closer in OSAS, and so also oropharynx and tonsils and fat pad and tonsils. In contrast, fat pad and oropharynx move farther apart from the skin object.

**Conclusions:**

The study has found several new anatomic bio-markers of OSAS. Changes in standardized T2-weighted image intensities in objects may imply that intrinsic tissue composition undergoes changes in OSAS. The results on inter-object distances imply that treatment methods should respect the relationships that exist among objects and not just their size. The proposed method of analysis may lead to an improved understanding of the mechanisms underlying OSAS.

## Introduction

### Background

The prevalence of childhood obesity has more than doubled in young children and quadrupled in adolescents in the past 30 years [1, 2]. In 2012, more than one third of children and adolescents were overweight or obese [3]. Children who are obese are at greater risk for obstructive sleep apnea syndrome (OSAS) and cardiovascular and metabolic disorders [4]. Understanding OSAS is particularly important, since it is also associated with neurocognitive and behavioral deficits and has shown to independently increase the risk for cardiovascular and metabolic derangements such as hypertension and insulin resistance [5]. In spite of the development of various diagnostic and therapeutic methods for OSAS [1, 6], the mechanisms contributing to OSAS in obese children still remain poorly understood.

Two types of modeling approaches have been employed to study OSAS–synthesis and analysis. In *synthesis*, a patient-specific biomechanical model of the upper airway is established [7, 8] to simulate airway dynamics by making use of the anatomic information derived from patient images. The model parameters and behavior are used to characterize OSAS. Its effectiveness depends on the data used for building the model and the fidelity of the model. In *analysis*, patient image data are processed to harness optimally OSAS-specific information that may reside in the images [6]. Its effectiveness also depends on the information content of the image data and the processing methods. There has been considerably more focus on the former approach in the literature than the latter. This paper concerns a method of analysis where we distill a collection of image measurements to arrive at a small set of potent markers of OSAS.

### Related research

A variety of imaging modalities have been utilized for quantitative analysis such as computed tomography (CT) [9–12], optical coherence tomography (OCT) [13, 14], and magnetic resonance imaging (MRI) [1, 6, 8, 15–20]. While OCT offers high spatial and temporal resolution, it is intrusive, has poor depth of penetration, and has shadowing effects. CT affords good spatial and reasonable temporal resolution but has poor contrast resolution for soft tissue structures and has radiation exposure concerns especially for imaging children. MRI, therefore, seems to be the ideal modality for studying OSAS at present. Measurements derived from MRI utilized in previous studies include upper airway cross-sectional area at specific locations [16, 17, 21, 22], upper airway volume/space [6, 23, 24], airway diameter change along the longitudinal direction, airway collapsibility [25], texture of airway muscle [26], and combinations of some of these features [27–29]. Many past efforts [6, 21–23] focused on single features derived from single objects, such as volumetric information for upper airway [6] or fat pad [23] where controls were weight-matched to the OSA group. Multiple features from single objects, such as size, cross-sectional area, and volume of upper airway have also been used together to characterize different patient groups [22, 25, 27–29]. For example, adult patient’s lesion length, location, and cross-sectional area are used in [22]; both the cross-sectional areas of the lower part of the pharyngeal airway and the volume of the upper part of the pharyngeal airway were analyzed in adults with anterior position of the mandible in [29].

Image analysis in previous research in OSAS has focused on just the upper airway or a couple of specific objects in its immediate vicinity, most commonly the adenoid and tonsils. The parameters (also referred to as *features* in this paper) studied were direct measures, such as volumes of objects, which were strongly suspected to be indicative of or causing OSAS. In this paper, we take a different approach–one that is broader and more general, of considering all major objects in the upper airway region, their individual morphological properties, their tissue characteristics revealed by image intensities, and the 3D architecture of the object assembly. From this fairly large collection of resulting parameters, we extract a few salient parameters that have the most discriminative and predictive value. We postulate that such an approach may shed new light on OSAS without being restricted by our guess of potentially important markers and studying only them in isolation. The parameters we study include object-specific size, surface area, volume, shape, and image intensity properties, as well as inter-object relationships and the correlation among object-specific measures. We quantitatively analyze these properties on 14 major structures in the upper airway region involving 159 parameters in total. The complete methodology, described in Section 2, consists of several image processing operations applied to the MRI data, deriving the full set of parameters, and subsequently selecting a small subset of them that exhibit negligible inter-parameter correlation and an ability to distinguish between the two subject groups. The results presented in Section 3 examine closely the predictive ability of the selected parameters to indicate subject group. Discussion as well as our conclusions are summarized in Section 4. Very early results covering some parts of this study were presented in the proceedings of the SPIE Medical Imaging 2015 conference [30].

## Materials and Methods

Our methodology consists of the following 6 main steps: (1) Establishing study groups and polysomnography data analysis. (2) Acquiring image data. (3) Defining and delineating objects in the images. (4) Deriving the full set of features. (5) Selecting a small subset of salient features. (6) Testing the ability of the selected features to predict patient groups. These steps are described below in detail.

### (1) Establishing study groups and polysomnography data analysis

Institutional Review Board approval was obtained for this study from Albert Einstein College of Medicine and Children’s Hospital at Montefiore and from the University of Pennsylvania. Written informed consent was obtained from next to kin, caretakers, or guardians on behalf of children enrolled in the study. The image data sets utilized in this study pertain to thirty subjects including male and female children. All subjects, 8–17 years of age (body mass index (BMI) > 95th percentile for age), were recruited from the adolescent, endocrine, and general pediatric obesity clinics at the Children’s Hospital at Montefiore as part of a larger study evaluating the pathophysiology of OSAS in obese children. All subjects had normal development and intact adenoid and tonsils. Diagnosis of OSAS or non-OSAS was based on the results of an overnight polysomnography. Accordingly, diagnosis of OSA was made in subjects with an apnea index (AI) > 1 event/hour and/or apnea- hypopnea index (AHI) > 5 events/hour. Tables 1 and 2 list the demographics and polysomnography data, respectively. Each group of OSAS and controls included 15 subjects. The OSA group age was 13.9 ± 2.0 years (mean ± SD), BMI was 36.0 ± 7.5 kg/m^2^, and AHI was 14.4 ± 10.7 events/hour. Controls’ age was 13.5 ± 2.7 years, BMI was 33.7 ±5.6 kg/m^2^, and the AHI was 1.0 ± 1.0 events/hour and was significantly lower than that of the OSA group (p < 0.001).

**Table 1:** Demographics of OSAS and Controls (Mean ± SD).

	OSAS (n = 15)	Controls (n = 15)	p Value
**Age (years)**	13.9 ± 2.0	13.5 ± 2.7	NS
**Gender (male/female)**	M:10, F:5	M:10, F:5	NS
**Height (cm)**	163.5 ± 13.6	160.8 ± 14.1	NS
**Weight (kg)**	96.9 ± 25.9	88.7 ± 24.3	NS
**BMI (kg/m**^**2**^**)**	36.0 ± 7.5	33.7 ± 5.6	NS
**BMI Z- Score**	2.4 ± 0.4	2.4 ± 0.3	NS

**Table 2:** Polysomnography of OSAS and Controls (Mean ± SD).

	OSAS (n = 15)	Controls (n = 15)	p Value
**Total Sleep Time (hrs)**	6.2 ± 1.6	6.3 ± 0.7	NS
**Sleep Efficiency (%)**	78.0 ± 17.5	84.9 ± 8.4	NS
**Apnea Index (events/hr)**	3.5 ± 5.8	0.3 ± 0.4	NS
**Apnea Hypopnea Index (events/hr)**	14.4 ± 10.7	1.0 ± 1.0	< 0.001
**Baseline SpO2 (%)**	98.9 ± 1.1	98.7 ± 1.2	NS
**SpO2 Nadir (%)**	85.5 ± 4.6	94.2 ± 2.7	< 0.001
**Baseline ETCO2 (mmHg)**	39.9 ± 6.8	41.7 ± 3.3	NS
**Peak ETCO2 (mmHg)**	50.5 ± 4.7	47.8 ± 3.9	NS
**Arousal Index (events/hr)**	14.0 ± 9.6	7.6 ± 3.8	0.03

### (2) Acquiring image data

The MR image data utilized in this investigation consist of axial T2-weighted and sagittal T1- and T2-weighted sequences. All acquisitions were on a Philips Achieva 3T machine with the following parameters. T2-weighted: TR/TE = 8274.3/82.6 msec, T1-weighted: TR/TE = 517.7/7.6 msec, image size 400 × 400 × 35–50, and voxel size 0.5 × 0.5 × 3.3 mm^3^. Data were obtained during tidal breathing in wakefulness in all subjects and during 2–3 minutes from multiple breaths (usually 10–18). The acquired images thus represent an averaged version of the airway and surrounding tissues during this period.

### (3) Object definition and image segmentation

For consistency and effectiveness of analysis, a definition of the neck body region was arrived at in terms of a starting and ending anatomic axial slice location for every subject–from the superior aspect of the eyes to 6.6 mm inferior to the inferior aspect of the mandible. In sagittal view, this definition was appropriately translated from the axial to the sagittal plane. All image data were clipped to match this definition of the body region. In the same way, each of the 14 objects considered in the study was consistently defined regarding its anatomy to specify what aspects of the object are considered for inclusion. Table 3 lists these objects, their definitions, and denotations.

**Table 3:** Objects in the upper airway region included in this study and their abbreviations and definitions.

Object	Abbreviation	Definition
**Skin**	sk	The outer boundary of the neck region skin. The interior region constitutes the entire neck body region and the object denoted Skin. The superior boundary is defined by the superior aspect of the eyes. The inferior boundary is defined by 2 slices (6.6 mm) inferior to the inferior aspect of the mandible.
**Pharynx & mandible**	pm = mn + ph	Grouping of mn and ph.
**Mandible**	mn	The outer boundary of the mandible.
**Pharynx**	ph = np + op	Grouping of np and op.
**Nasopharynx**	np	This pharynx subregion is defined by the airways, where the inferior boundary is defined by the inferior aspect of the soft palate.
**Oropharynx**	op	This pharynx subregion is defined by the airways, where the inferior boundary is defined by the superior aspect of the epiglottis and superior boundary by the inferior aspect of the soft palate.
**Fat pad**	fp	The outer boundary of the lateral pharyngeal fat pad.
**Adenoid & Tonsils**	at = tn + ad	Soft tissue grouping of tn and ad.
**Tonsils**	tn = tR + tL	Grouping of tR and tL.
**Right Tonsil**	tR	The outer boundary of the right tonsil.
**Left Tonsil**	tL	The outer boundary of the left tonsil.
**Tongue**	tg	The outer boundary of the tongue, segmented in the sagittal view.
**Soft Palate**	sp	The outer boundary of the soft palate, adjacent and posterior to the hard palate.
**Adenoid**	ad	The outer boundary of the adenoid.

**Fig 1:**
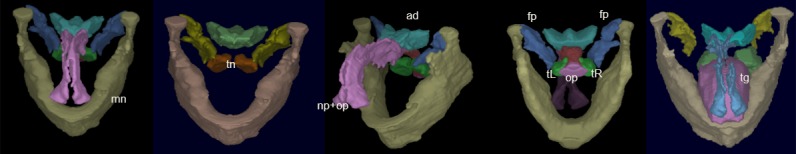
3D surface renditions of some segmented objects from one subject. Objects shown in different combinations are: np (nasopharynx), op (oropharynx), mn (mandible), tn (tonsils), tL & tR (left and right tonsil), fp (fat pad), ad (adenoid), tg (tongue).

For each object, the 3D region enclosed by the boundary surface was considered to represent the object. Each object was carefully delineated under close human supervision in all 30 image sets by using a combination of image segmentation tools [31] implemented in the CAVASS software system [32]. All results were checked for accuracy via 3D surface renditions of each object separately and in different combinations with other objects. Surface renditions of some representative objects are shown in Fig 1. The 3D renditions provide a quick check for object consistency and any errors in segmentation in the form of object discontinuities from slice to slice. When errors were found through this scrutiny, they were verified via slice displays of the delineations overlaid on the slices and were subsequently corrected.

Features derived from the object assembly include those specific to the individual objects as well as relationships among objects. These are described in the following sections.

### (4) Deriving features

Object-specific features considered include linear size, volume, surface area, sphericity, and standardized image intensity value. Inter-object relationships considered were the inter-object distances for all possible pairs of objects.

Size (*S*_*λ*_): The idea behind a size estimate for an object is to express a measure of the “largeness” of the object by a single number. We define the size of an object by $\sqrt{\left(\lambda_{1}+\lambda_{2}+\lambda_{3}\right)},$ where *λ*_1_, *λ*_2_, and *λ*_3_ are the eigenvalues obtained by principal component analysis of the entire 3D object region. Principal component analysis [33] is a commonly-used statistical pattern recognition procedure that employs an orthogonal transformation to convert a set of observations of possibly correlated random variables into a set of values of linearly uncorrelated random variables called principal components. Roughly speaking, the eigenvalues resulting from applying this analysis to the points in the 3D object region indicate the variance (dispersion) of the object points in the three directions represented by the corresponding eigenvectors. The largest eigenvector indicates the direction of elongation of the object and the other two eigenvectors indicate the direction of the breadth and thickness of the object. The size parameter roughly corresponds to the sum of the major and minor axes lengths of an ellipsoid that approximates the object.

Normalization of size: The size described above is an absolute measure. We considered it important to normalize this size to account for variation in the physical size of individual subjects. It may be expected that larger individuals will have overall larger organs. The factor of normalization we employed was the length of the diagonal of a box that just encloses the mandible, the idea being that the mandible partially encloses the upper-airway region, constraining other objects, and may be a good indicator of the overall physical size of a subject. If *L* denotes this normalizing length for a subject, then the normalized size measure of an object O is expressed as $S_{\lambda }\left(\text{O}\right)=\sqrt{\left(\lambda_{1}+\lambda_{2}+\lambda_{3}\right)}\;/\;L.$ The same normalization operation is also applied to the measurements of volume and surface area, and as such these three measures (size, volume, surface area) are unit-less. We note that in previous OSAS studies involving size measures, no normalization was considered.

Volume (*S*_*V*_): The volume *S*_*V*_(O) enclosed by the surface of an object O is computed by first representing the object surface as a triangle mesh from the given 3D binary image of the object and then carrying out digital surface integration [34]. We define the normalized volume measure as *S*_*V*_(O) = (Volume of O) /*L*^3^.

Surface area (*S*_*A*_): The surface area of an object O is computed from its triangular surface representation as the total area of the triangles [34]. We define the normalized surface measure as *S*_*A*_(O) = (Surface area of O) /*L*^2^.

Sphericity (*S*_*P*_): This is a unit-less measure which expresses object shape complexity as compared to a sphere. The formula given below, which is dependent on volume *S*_*V*_ and surface area *S*_*A*_ of the object, is derived in such a manner that for a sphere this measure takes on a value of 1. All other shapes will have higher values. It expresses how an object O deviates in shape from being spherical. Note that different shapes can still have the same sphericity value.

$$S_{P}(\text{O})=\frac{\sqrt[{3}]{36\;\pi \;S_{V}^{2}(\text{O})}}{S_{A}(\text{O})}.$$ (1)

Standardized image intensity values (*H*_*I*_): Image intensities in MRI usually do not have a tissue-specific numerical meaning. That is, even when the same subject is scanned on the same scanner repeatedly for the same body region under same conditions using the same imaging sequence, the image intensity values in the same tissue region may differ. We used the intensity standardization methods described in [35], which have been shown to achieve tissue-specific numeric meaning to overcome this problem. Our goal was to study if tissue-specific MRI properties differed in the two groups of subjects. It has been demonstrated that, by using intensity standardization, studies comparing different subject groups based on tissue properties characterized by image intensities can be carried out [36]. *H*_*I*_(O) denotes the mean standardized image intensity over all voxels within the object O under consideration. The standardized intensity scale was arbitrarily set to the range [0, 4095] for all objects and all MRI imaging sequences. In the methods of [35], this scale can be set at the time of initial calibration of the intensities.

Inter-object distances (*d*(*A*, *B*)): The geometric center of each object is taken to be its reference point, and the distance *d*(*A*, *B*) between any two objects *A* and *B* is defined as the Euclidean distance between their geometric centers normalized by *L*: *d*(*A*, *B*) = (Distance between *A* and *B*)/*L*. This measure is also unit-less.

In summary, we had 14 objects which yielded 91 inter-object distances (14 choose 2 = 14 × 13 /2). Each object had 5 object-specific features (size, volume, surface area, sphericity, standardized image intensity) yielding 14 × 5 = 70 features. However, *H*_*I*_ values for tongue and soft palate were excluded since these objects were impossible to segment reliably on the axial T2-weighted images, and were delineated on the sagittal T1-weighted images in some cases and on the T2-weighted images in other cases. Thus the total number of features was 91 +70–2 = 159.

### (5) Selecting salient features

Our goal was to select from the full set F of *M* features (in our case *M* = 159) a smaller set ϕ of *N* features from the full set F such that *N* is much smaller than *M*. Various feature transform techniques are available in the pattern recognition literature, such as principal component analysis. We are interested in those techniques that derive the smaller set ϕ as truly a subset of the full set F rather than by transforming F such that the new features are defined in a different mathematical space whose meaning is lost from the original measurements. If the feature space changes, then it becomes impossible to interpret the meaning of the new features since they lose the physical meaning carried by the original features in F. Maintaining the original meaning is important from the perspective of the diagnostic and therapeutic use of the selected features.

Our approach to derive the smaller set ϕ from the full set F consists of three steps. In the first step, a subset U of F is found such that U consists of those features of F which have no greater than a low degree (-ve or +ve) of correlation with most other features in F. It is generally difficult to guarantee that any feature in F is truly independent, as a random variable, of all other features in F. Therefore, we adopted a weaker condition of accepting a low degree of correlation with a sufficiently large number of other features of F as a reasonable and practical alternative. Accordingly, to implement this strategy, we introduce two variables–a correlation interval limit [-δ, δ], and a lower limit, denoted by variable *M*_*L*_, to indicate the number of features with which we require a feature to have low correlation. In words, the idea is that for a feature to qualify as a member of set U, it should have low correlation within the interval [-δ, δ] with at least *M*_*L*_ features of F. Note that the size of set U depends on both δ and *M*_*L*_.

In the second step of computing ϕ from F, a separate subset Q of F is determined such that the features in Q are capable of separating the two patient groups with statistical significance. In the third step, the set ϕ of finally selected features is determined as the intersection of the above two sets, ϕ = U ∩ Q. Thus the features in ϕ are those features in F which are mostly uncorrelated (or have negligible correlation among themselves) and which are able to separate the two patient groups.

### (6) Testing predictability

The features in ϕ are used to test their ability to correctly classify (predict) a new patient image into one of the two patient groups by using logistic regression based on a sigmoid model. Given a feature set ϕ, classification performance is tested by using the leave-n-out (n > 1) strategy by randomly selecting n out of a total of m test images, using m-n samples for training the classifiers, and n samples to test them. This process is repeated 30 times. In our case, m = 30 and n = 6. For all 30 experiments, the feature set ϕ is fixed once selected. Only the classifier parameters may change from one experiment to the next because of the different training samples used in each experiment.

## Results

### Salient features

**Fig 2:**
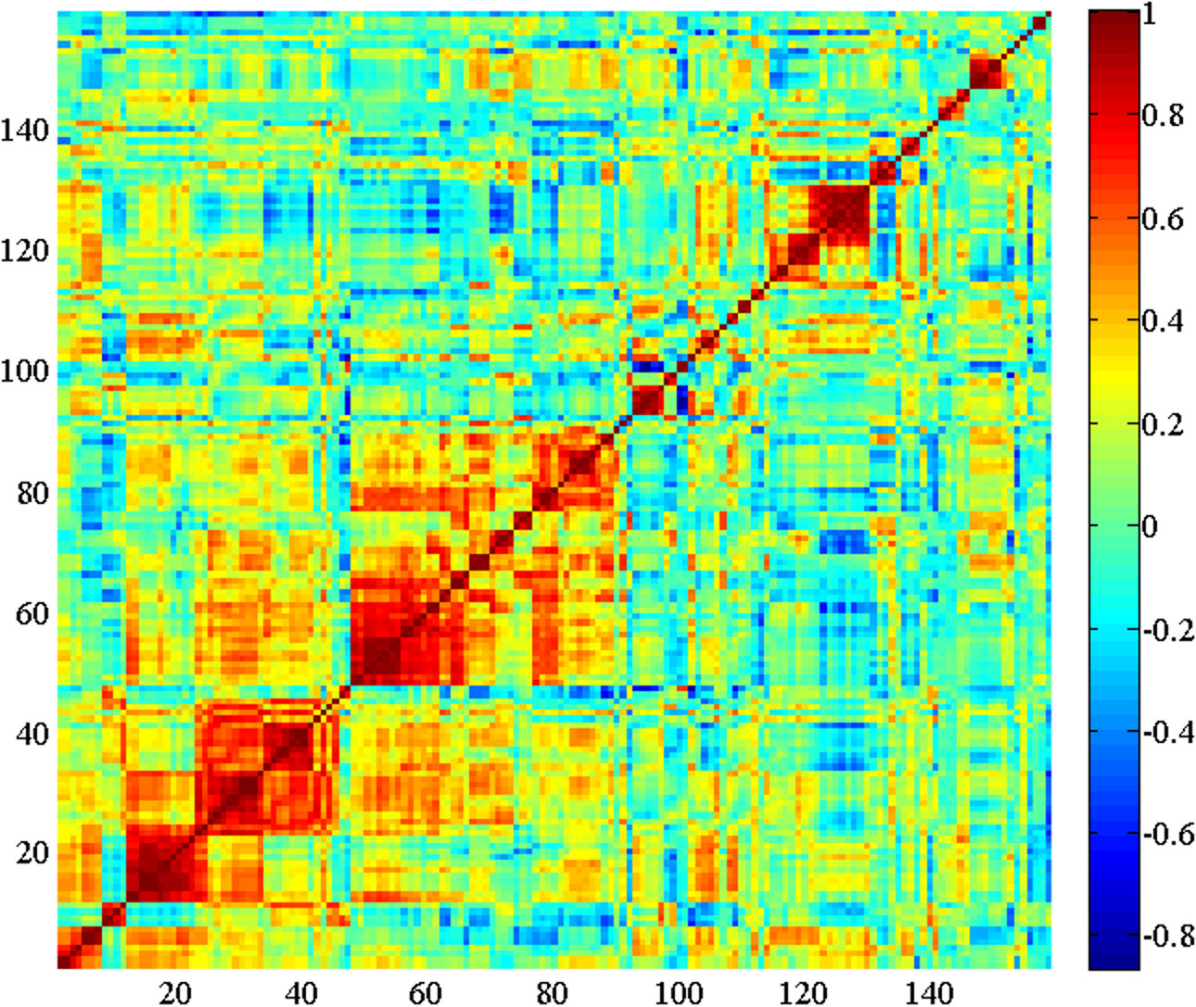
Heat map representation of correlation between all possible pairs of features. Horizontal and vertical axes represent features.

**Fig 3:**
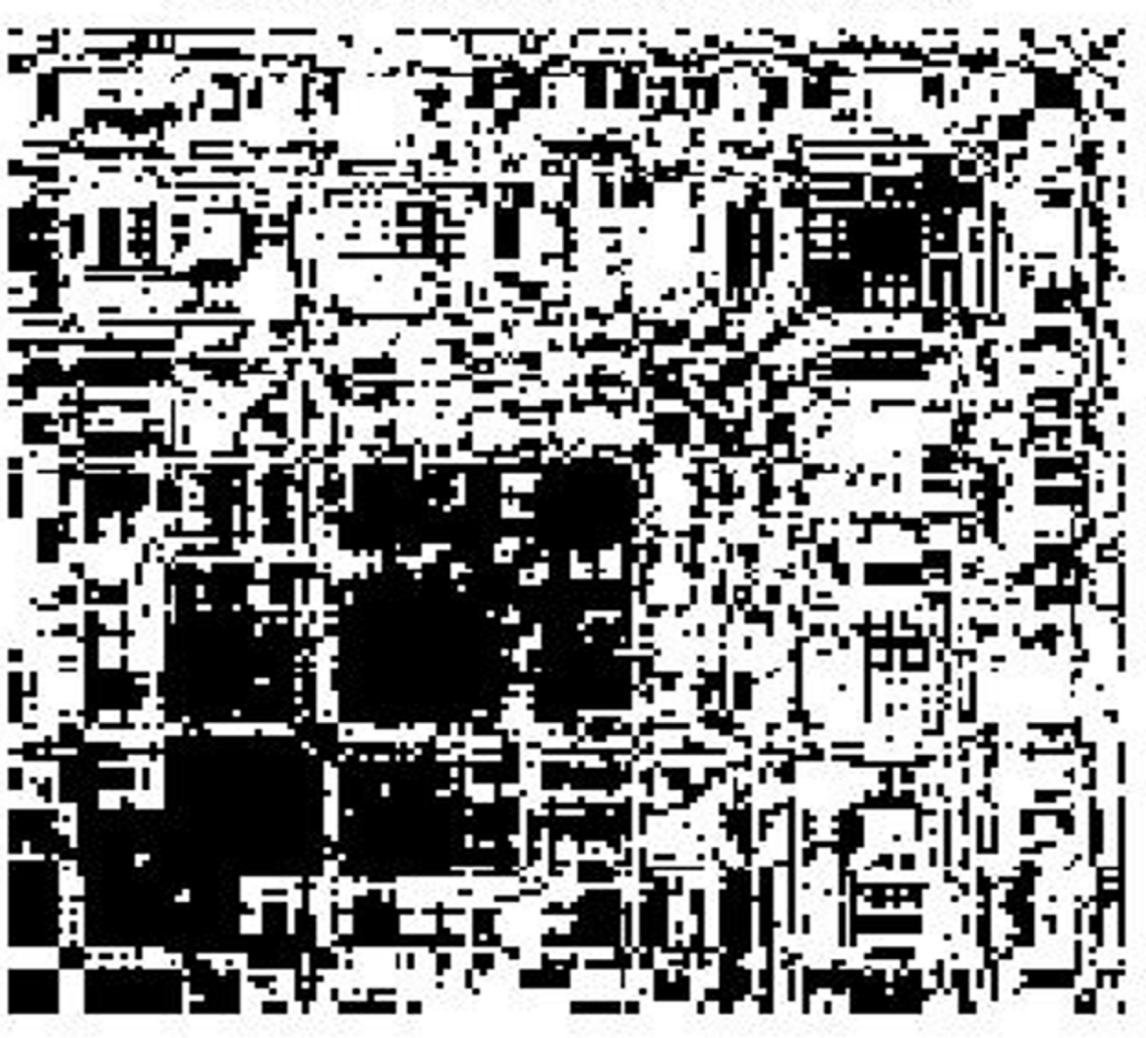
Heat map of Fig 2 after thresholding. Correlations falling within [-0.2, 0.2] are shown in white.

For a visual interpretation of the correlations among features, we use a color graphical method called *heat map* to represent the correlation matrix. Thus the horizontal and vertical axes of the map represent the features. In the heat map visualization method, the columns and rows of the correlation matrix are organized by similarity in such a way that similar behaving rows and columns are clustered by using an automatic clustering technique. The advantage of the heat map method over simple numerical correlation matrices is better global visualization of the information in an organized and readily perceptible manner, especially when the number of features involved is large. The intensity of the correlations is expressed on a heated scale from dark blue to dark red, where blue corresponds to negative correlations and red corresponds to positive correlations. Fig 2 shows the heat map display of the correlations among all 159 features derived from all data from both subject groups pooled together. The heat map shows several strongly positively correlated (red) regions and fewer strongly negatively correlated (blue) regions. Because of the difficulty of showing the names of actual features in a limited space, we do not indicate feature names along the x and y axes. As an example, the square deep red region with coordinates of its diagonally opposite corners roughly at (11, 11) and (22, 22) represents the feature set {*d*(op, ph), *d*(sk, mn), *d*(sk, pm), *d*(sk, tL), *d*(sk, tn), *d*(sk, tR), *d*(sk, tg), *d*(sk, sp), *d*(sk, at), *d*(sk, fp)}, which corresponds to mostly normalized distances between skin and other objects. In Fig 3, we display a pixel in white when the correlation between the corresponding pair of features falls within the threshold interval [-0.2, 0.2]. This picture is essentially the result of thresholding the heat map for this threshold interval. Clearly the number of features that are uncorrelated or have low levels of correlation is large.

**Fig 4:**
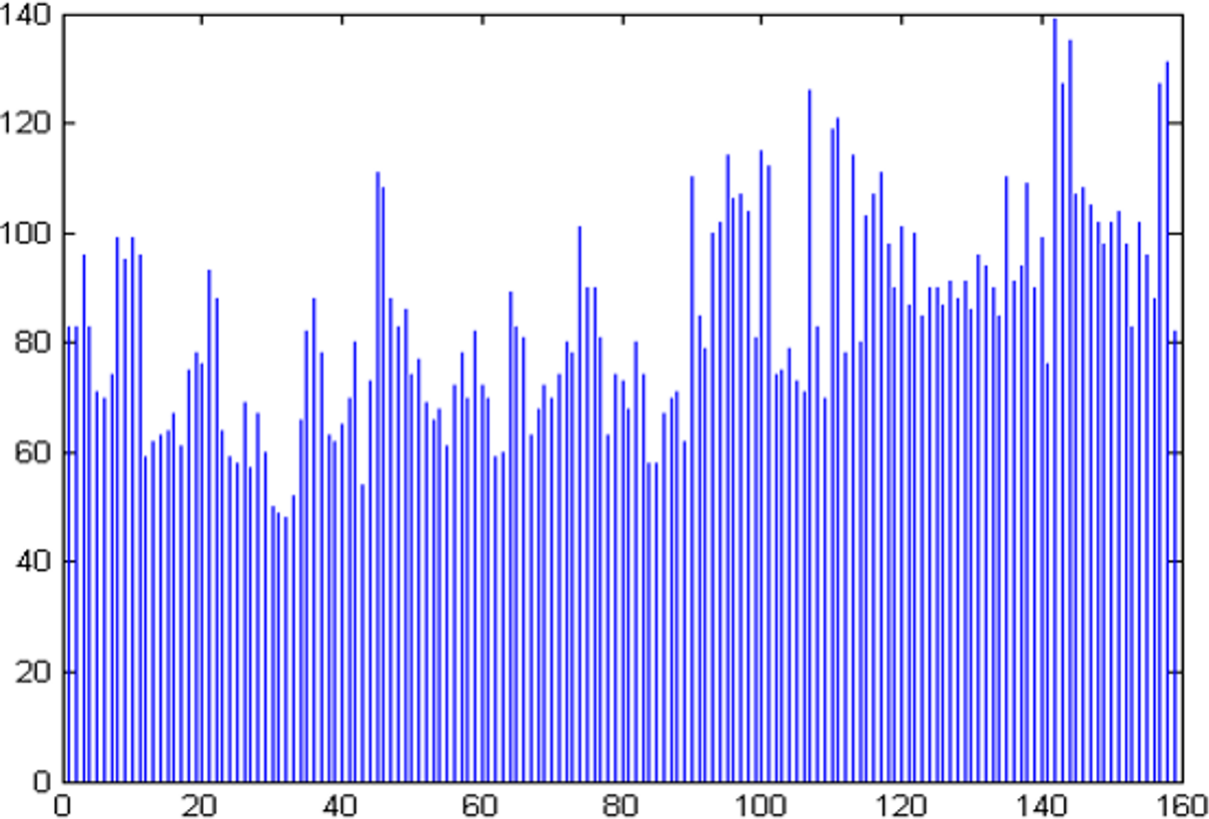
A plot of number of features with low correlation in [-0.2, 0.2] with respect to each feature. The horizontal axis denotes the features in set F and the vertical axis denotes the number of features in F with which the selected feature on the horizontal axis has low correlation.

Fig 4 is a plot of the number of features that have low levels of correlation (within [-0.2, 0.2]) with respect to each feature. In this graph, the horizontal axis represents the 159 features and the vertical axis denotes the number of features with which the selected feature on the horizontal axis has low correlation. For computing U, we set *M*_*L*_ = 100 and δ = 0.2. These values were selected experimentally. We first chose M_L_ close to M and then adjusted the value of δ until the resulting number of features was in the teens. As we will see below, we actually searched over the full range of values [-δ, δ] = [-1, 1] in order to identify set ϕ that yielded the best accuracy for patient group prediction, and so the range [-0.2, 0.2] was used only for the illustration in Fig 4.

**Fig 5:**
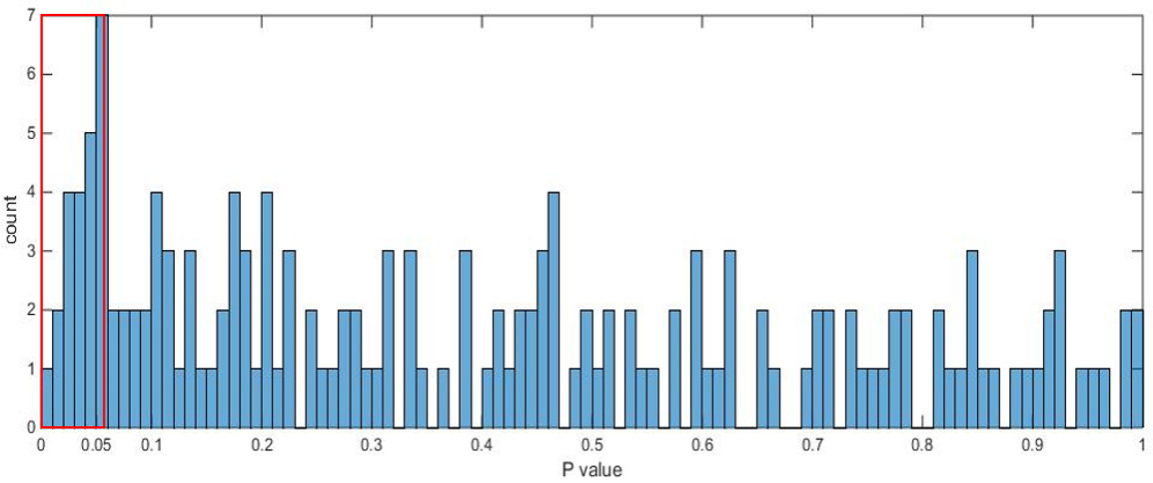
Distribution of *p* values from T-tests carried out for each feature for group separability.

To examine if the *p* values from the T-tests (two-tailed and unpaired samples with unequal variance), which assess the ability of each feature on its own to separate the two subject groups, achieve significance by chance, we display in Fig 5 the distribution of the *p* values over all features. The histogram suggests that the significance achieved is not by chance and there are several features that achieve a value *p* < 0.05. In Table 4, such features and their mean and standard deviation values are presented.

**Table 4:** Mean and standard deviation of *S*_*λ*_, *S*_*V*_, *S*_*A*_, *S*_*P*_, *H*_*I*_, and *d*(*A*, *B*) for those objects which showed statistically significant differences between the two groups.

Feature	Object	Control		OSAS		*p*-value
		Mean	Std deviation	Mean	Std deviation	
***S***_***λ***_	sk	0.429	0.021	0.445	0.022	0.052
	ad	0.072	0.009	0.079	0.010	0.048
***S***_***V***_	at	0.003	0.001	0.004	0.002	0.054
	tn	0.0017	0.001	0.0021	0.001	0.054
	sk	0.630	0.092	0.705	0.101	0.043
***S***_***A***_	sk	5.089	0.427	5.512	0.577	0.031
	at	0.286	0.068	0.340	0.072	0.046
	fp	0.225	0.038	0.279	0.074	0.020
	ad	0.139	0.038	0.171	0.051	0.034
***S***_***P***_	fp	0.297	0.023	0.280	0.019	0.032
	op	0.495	0.058	0.450	0.050	0.034
***H***_***I***_	sk	1233.5	40.2	1269.9	42.0	0.022
	ph	296.5	44.1	262.2	47.2	0.049
	fp	2423.9	139.8	2352.9	81.7	0.052
	np	328.6	56.9	278.9	56.6	0.023
***d*(*A*,*B*)**	sk, op	0.183	0.034	0.220	0.044	0.016
	sk, fp	0.089	0.035	0.129	0.055	0.026
	mn, np	0.250	0.030	0.228	0.028	0.055
	op, tL	0.095	0.014	0.082	0.010	0.007
	fp, tg	0.309	0.026	0.290	0.022	0.042
	fp, tL	0.161	0.022	0.143	0.022	0.018
	tL, tR	0.154	0.027	0.135	0.025	0.054
	tR, tg	0.207	0.018	0.194	0.014	0.035

Features in the intersection set ϕ are listed in Table 5 for values of δ = 0.15, 0.2, 0.25, and 0.3. Note that ϕ becomes empty for δ ≤ 0.15. Also, clearly features selected at a lower value of δ are included among features selected at a higher δ value. Thus, in Table 5, ϕ grows from left to right in the last row, by adding new elements as the value of δ increases.

**Table 5:** Salient features selected by the method and the associated parameters.

**δ**	**0.15**	**0.2**	**0.25**			**0.3**		
**Cardinality of U**	7	33	71			112		
**Cardinality of Q**	16	16	16			16		
**Cardinality of ϕ**	0	2	6			13		
**ϕ**	** **	*S*_*V*_(sk)	*H*_*I*_(ph)	*S*_*V*_(sk)	*S*_*A*_(sk)	*S*_*ϕ*_(ad)	*H*_*I*_(sk)	*H*_*I*_(ph)
						*H*_*I*_(np)	*S*_*V*_(sk)	*S*_*A*_(sk)
						*S*_*p*_(fp)	*S*_*p*_(op)	*d*(fp, tg)
						*d*(sk, op)	*d*(sk, fp)	*d*(op, tL)
		*d*(fp, tg)}	*d*(sk, op)	*d*(sk, fp)	*d*(fp, tg)	*d*(fp, tL)		

### Prediction from logistic regression

Classification accuracy derived from the 30 experiments based on logistic regression is summarized in Table 6 for different sizes of the set ϕ of salient features. Clearly, by selecting ϕ as indicated in Table 5, it is possible to order all 159 features by “importance”. Table 6 shows how prediction accuracy changes as we recruit more and more features into set ϕ. The highest accuracy of close to 96% is achieved when ϕ had 16 features corresponding to δ = 0.36. These features are enumerated in Table 7 along with their group-wise mean and standard deviation values.

**Table 6:** Prediction accuracy by logistic regression.

Number of features in ϕ	6	10	13	16	30	50	80	120	159
**Accuracy (%)**	63.9	83.9	84.4	96.1	85.0	76.1	72.2	65.6	55.0
**TP (%)**	64.4	84.4	78.9	97.8	91.1	77.8	75.6	65.6	56.7
**FP (%)**	36.7	16.7	10.0	5.6	21.1	25.6	31.1	34.4	46.7

**Table 7:** The 16 optimal features forming set ϕ obtained for δ = 0.36, their description, and their group-wise mean and standard deviation.

Feature description	Feature	Control	OSAS	*p*
**Size of adenoid**	*S*_*λ*_(ad)	0.072 (0.009)	0.079 (0.010)	0.048
**Volume of skin object**	*S*_*V*_(sk)	0.630 (0.092)	0.705 (0.101)	0.043
**Surface area of skin object, adenoid & tonsils, fat pad**	*S*_*A*_(sk)	5.089 (0.427)	5.512 (0.577)	0.031
	*S*_*A*_(at)	0.286 (0.068)	0.340 (0.072)	0.046
	*S*_*A*_(fp)	0.225 (0.038)	0.279 (0.074)	0.020
**Sphericity of oropharynx, fat pad**	*S*_*p*_(op)	0.495 (0.058)	0.450 (0.050)	0.034
	*S*_*p*_(fp)	0.297 (0.023)	0.280 (0.019)	0.032
**Standardized intensity of pharynx, skin object, nasopharynx**	*H*_*I*_(ph)	296.5 (44.1)	262.2 (47.2)	0.049
	*H*_*I*_(sk)	1233.5 (40.2)	1269.9 (42.0)	0.022
	*H*_*I*_(np)	328.6 (56.9)	278.9 (56.6)	0.023
**Inter-object distance**	*d*(fp, tg)	0.309 (0.026)	0.290 (0.022)	0.042
	*d*(tR, tg)	0.207 (0.018)	0.194 (0.014)	0.035
	*d*(sk, op)	0.183 (0.034)	0.220 (0.044)	0.016
	*d*(sk, fp)	0.089 (0.035)	0.129 (0.055)	0.026
	*d*(op, tL)	0.095 (0.014)	0.082 (0.010)	0.007
	*d*(fp, tL)	0.161 (0.022)	0.143 (0.022)	0.018

**Fig 6:**
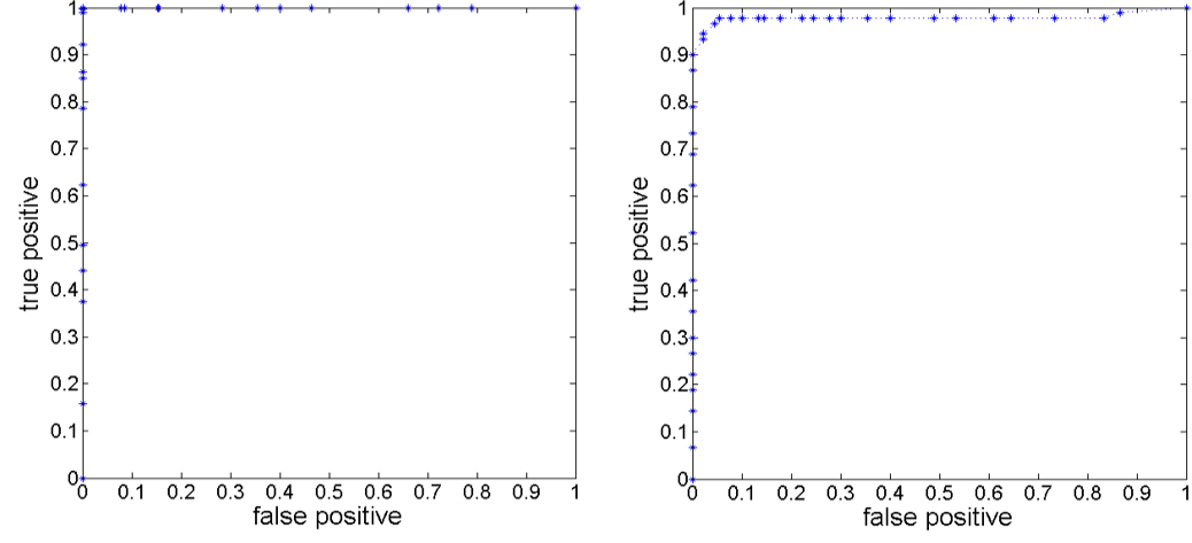
ROC curves for the classification task on the training samples (left) and on the testing samples in cross validation (right).

Fig 6 shows two receiver operating characteristic (ROC) curves, one for predicting on training samples and another for predicting on testing samples in the cross validation experiments by employing these 16 optimal features. Shrinkage of “Area under the curve” (AUC) values from training to testing samples is about 3%, the respective AUC values being 1 and 0.97.

## Discussion

In the specific area of image analysis in OSAS, most published papers are restricted to correlative studies of finding image features that correlate with clinical parameters, which in the case of OSAS are chiefly (AHI and oxygen desaturation index (ODI) [1, 28, 37, 38]. Reference [39] is an exception which proposed a predictive model for OSAS in obese adolescents by using a scoring system which is based on the sum of scores on tonsillar hypertrophy (T), adenoid hypertrophy (A), and neck circumference (NC). Adeno-tonsillar hypertrophy was determined based on paranasal sinus radiographic images. Its sensitivity (true positive) of diagnosing OSAS of 62% is much lower than the 96% achieved by our approach, and its specificity (true negative) of 100% is slightly better than our 94.5%.

More specifically, studies that focused on distinguishing between obese non-OSAS and OSAS groups have analyzed anatomic volumes [1, 6], object distances [27], cross-sectional areas of a couple of objects—mostly the airway, tonsils, and adenoid [16, 17, 21, 22]–and did not address the issue of finding biomarkers for class prediction. We took a general approach of considering a large collection of features which are potential markers of OSAS and then, through the proposed methods, distilled a small subset. The set of top 16 features listed in Table 7 yielded a remarkably high accuracy of class prediction, which is quite unusual in the general image analysis literature on finding biomarkers of diseases.

Several observations can be made by examining these 16 features to potentially gain new insight into OSAS. (1) Standardized T2-weighted image signal intensities differ between the two patient groups for the entire neck body region, pharynx, and nasopharynx, possibly indicating changes in object tissue characteristics. Such studies have not been done in the past. (2) Adenoid increases in linear size as do surface area and volume of the skin object (entire neck body region, see Table 3) and the surface area of adenoid, tonsils, and fat pad in OSAS. Object volume based comparisons were previously studied by others with similar observations [1, 6] for fat pad, adenoid and tonsils. The main difference in our study was that we normalized all geometric measurements with respect to *L*, the linear size of the mandible, to account for subject-to-subject body size variations. (3) Sphericity decreases in OSAS for fat pad and oropharynx, implying that these objects become less round or more complex in shape in OSAS. (4) In OSAS, some inter-object distances increase while others decrease. Specifically, fat pad and tongue move closer, and so also oropharynx and tonsils, and fat pad and tonsils. In contrast, fat pad and oropharynx move farther apart from the skin object.

This paper went beyond the object volume parameter that has been previously investigated and set out to study if the consideration of all major objects and a variety of object-specific and object assembly properties would be useful in characterizing OSAS in obese children. In this process, the paper makes two contributions. (1) It has found several new anatomic biomarkers of OSAS as summarized above. (2) The proposed method of analysis may lead to an improved understanding of the mechanisms underlying OSAS. For example, changes in standardized T2-weighted image signal intensities in objects may imply that intrinsic tissue composition undergoes changes in OSAS. The results on object distance relationship, especially among fat pad, tongue, airway, and tonsils, imply that treatment methods should take into account the relationships that exist among objects and not just their size.

The main limitation of this study is the small number of subjects studied. However, although some of the observations may change, the method itself is applicable to larger populations. It may be more useful if the set of features can be reduced from 16 to a much smaller number. It is interesting to note that accuracy jumped from 6 to 10 and 13 to 16 features but was negligible from 10 to 13 features. It is feasible to add a fourth step to our methodology of finding the most potent among the set ϕ of salient features selected, which may allow us to a smaller set of features for a given required level of accuracy. Note also that this is a general analytics methodology of image-based biomarker selection which is applicable to other body regions and disease processes.

The implications of the new observations in this paper for developing new treatment protocols require extensive further studies. However, the method and the results of the study can be immediately utilized to evaluate outcomes of current and new treatment methods in a more comprehensive manner.
